# A deep learning-based framework for retinal fundus image enhancement

**DOI:** 10.1371/journal.pone.0282416

**Published:** 2023-03-16

**Authors:** Kang Geon Lee, Su Jeong Song, Soochahn Lee, Hyeong Gon Yu, Dong Ik Kim, Kyoung Mu Lee

**Affiliations:** 1 Department of Electrical and Computer Engineering, ASRI, Seoul National University, Seoul, South Korea; 2 Department of Ophthalmology, Kangbuk Samsung Hospital, Sungkyunkwan University School of Medicine, Seoul, South Korea; 3 Biomedical Institute for Convergence (BICS), Sungkyunkwan University, Suwon, South South Korea; 4 School of Electrical Engineering, Kookmin University, Seoul, South Korea; 5 Sky Eye Clinic, Seoul, South Korea; 6 HanGil Eye Hospital, Incheon, South Korea; 7 Interdisciplinary Program in Artificial Intelligence, Seoul National University, Seoul, South Korea; Politechnika Slaska, POLAND

## Abstract

**Problem:**

Low-quality fundus images with complex degredation can cause costly re-examinations of patients or inaccurate clinical diagnosis.

**Aim:**

This study aims to create an automatic fundus macular image enhancement framework to improve low-quality fundus images and remove complex image degradation.

**Method:**

We propose a new deep learning-based model that automatically enhances low-quality retinal fundus images that suffer from complex degradation. We collected a dataset, comprising 1068 pairs of high-quality (HQ) and low-quality (LQ) fundus images from the Kangbuk Samsung Hospital’s health screening program and ophthalmology department from 2017 to 2019. Then, we used these dataset to develop data augmentation methods to simulate major aspects of retinal image degradation and to propose a customized convolutional neural network (CNN) architecture to enhance LQ images, depending on the nature of the degradation. Peak signal-to-noise ratio (PSNR), structural similarity index measure (SSIM), *r*-value (linear index of fuzziness), and proportion of ungradable fundus photographs before and after the enhancement process are calculated to assess the performance of proposed model. A comparative evaluation is conducted on an external database and four different open-source databases.

**Results:**

The results of the evaluation on the external test dataset showed an significant increase in PSNR and SSIM compared with the original LQ images. Moreover, PSNR and SSIM increased by over 4 **dB** and 0.04, respectively compared with the previous state-of-the-art methods (*P* < 0.05). The proportion of ungradable fundus photographs decreased from 42.6% to 26.4% (*P* = 0.012).

**Conclusion:**

Our enhancement process improves LQ fundus images that suffer from complex degradation significantly. Moreover our customized CNN achieved improved performance over the existing state-of-the-art methods. Overall, our framework can have a clinical impact on reducing re-examinations and improving the accuracy of diagnosis.

## Introduction

Retinal fundus photography is an invaluable examination tool in ophthalmology for diagnosing and monitoring retinal disease. It is important because of its reliability, non-invasiveness, low maintenance, and inexpensiveness. It enables clinicians to observe the retina in detail through high-quality and high-resolution images. Retinal fundus photography is one of the most basic imaging modalities, and it is used to diagnose major retinal diseases, such as age-related macular degeneration and diabetic retinopathy.

An increase in life expectancy globally [1] is likely to increase chronic age-related eye diseases. Thus, the demand for high-quality fundus photography is expected to rise accordingly. In the Republic of Korea, regular systemic health screening is mandatory for adults 40 years and above. In 2015, 76.1% of adults in this age category received an annual health examination (National Health Screening Statistical Yearbook, National Health Insurance Corporation, 2016) [2], and fundus photography was one of the optional screening tools.

Despite that the retinal cameras used for eye screening achieve state-of-the-art technology for fundus images, the quality of each fundus image may vary depending on the environment, the operator, or the patient. For instance, motion blur can occur if the patient moves, or the image may contain occlusions or have insufficient illumination if the patient blinks. Thus, the clinician may face challenges in conducting an effective diagnosis, and the issue may make these fundus images ungradable. In this case, the patients must be re-examined to acquire accurate fundus photography results, leading to unnecessary costs and time delay.

Recently, deep learning models have had a huge impact on image classification [3, 4], image segmentation [5, 6], and successful application to retinal fundus images [7–10]. Many deep-learning models have also been proposed to improve degraded images. Convolutional neural networks (CNN) for image and video deblurring [11–13] and super-resolution [14–16] have achieved state-of-the-art performance. CNNs are trained in a supervised learning framework, depending on the training images and their corresponding ground truth (GT) images. Training pairs of low-quality (LQ) and high-quality (HQ) images are vital to developing a CNN model for fundus image enhancement.

However, it is very difficult to physically construct a dataset of corresponding training images because it is difficult to control or reproduce complex image degradation. Several datasets for image enhancement have been collected manually [17] or by synthesizing a particular image degradation [18, 19]. Previous studies that synthesized training images tended to model only a single aspect of image degradation [20, 21]. However, simulating the compounded factors into complex degradation is challenging.

Thus, we developed a new deep learning-based model to enhance LQ retinal fundus images that suffer from complex degradation. Specifically, we developed a new supervised learning framework, comprising new processes for dataset construction, data augmentation, and a new model for supervised learning. To this end, we established a process to construct a dataset of LQ and HQ image pairs.

LQ images contain various degradation, such as blur, haze, low illumination, and artifacts such as eyelashes or tears. Moreover, we include various abnormal images with diseases and normal images without disease within the LQ and HQ image pairs so that the framework is unbiased toward normal images. Based on this dataset, we propose a framework for data augmentation and a novel CNN structure that can enhance images depending on the degradation. We conducted comparative quantitative and qualitative evaluations using private and public datasets to demonstrate the effectiveness of the proposed method.

Overall, our main contributions are as follows:

- We establish a unique training dataset that includes LQ and HQ image pairs, consisting of various abnormal features for major eye diseases, which differs from that of other studies that apply a single diagnosis (for example, diabetic retinopathy). We trained the framework to preserve all the clinically important features during the enhancement process because approximately 50% of our dataset has at least two or more diagnoses of diseases such as age-related macular degeneration, diabetic retinopathy, and epiretinal membrane.
- We propose data augmentation methods to simulate major aspects of retinal image degradation, including blur, haze, and low illumination to reduce the limitations in the dataset collection.
- We present a customized CNN architecture that incorporate attention layers into the U-net structure, resulting in improved performance in quantitative and qualitative evaluations.

## Related works

### Deep learning-based methods for retinal fundus images

Recently, advanced deep learning-based systems have achieved significant performance in the grading and classification of retinal fundus images and in detecting specific landmarks (mainly vessels) or diseases, such as diabetic retinopathy.

Several works [22–25] have proposed automatic retinal fundus image grading systems using a CNN as the backbone to generate feature vectors that are given as the input of a classifier. These methods may be the basis of more automated clinical procedures compared to existing traditional procedures for retinal diseases where doctors performed the jobs manually. A study [26] has shown that the extracted retinal image feature can be used as an input for recurrent neural networks to generate a detailed clinical description.

Many recent studies have stressed that using simple CNN architecture to extract features from retinal fundus images can effectively improve the performance of the vessel segmentation task [27–31]. Other studies [32, 33] proposed to apply dilated convolution to overcome the limited information with a fixed-sized receptive field of conventional CNN architectures to better estimate the vessels in the retinal fundus image. In the work of Jiang et al. [34], a multiscale information fusion module is added to the dilated CNN architecture to enlarge the receptive field of the CNN.

Some studies have shown the effectiveness of using attention mechanisms with multiscale operations or enlarged receptive fields. Zhang et al. [35] proposed an attention-guided filter to recover spatial information and merge structural information from the various resolution levels by filtering the low-resolution feature maps with high-resolution feature maps. Jiang et al. [36] also proposed a residual attention module to highlight important areas in fundus images, filter noise from the background, and solve the problem of information loss caused by down-sampling. In Mou et al. [37], both the 2-dimensional spatial attention and channel attention modules were used to enrich contextual dependencies over local feature representations, and exploit the interdependencies of channel maps, resulting in improved vessel segmentations.

Many other studies have particularly based on the U-Net [38] architecture. Gao et al. [39] formulated the vessel segmentation task as a multi-label problem and combined the Gaussian matched filter with U-Net to generate a blood vessel segmentation framework. Alom et al. [40] proposed the Recurrent CNN (RUNet) and Recurrent Residual CNN model (R2U-Net) architectures for segmentation tasks. Kamran et al. [41] proposed a multiscale generative architecture for accurate retinal vessel segmentation and to alleviate the inability of the decoder to recover lost information from the encoder of the U-Net.

### Enhancement of retinal fundus images

Several methods have been proposed that recover details of the vessels or the macula from degraded LQ images by enhancing the brightness, contrast, or luminance of images. Zhou et al. [42] and Palanisamy et al. [43] revealed that luminance and contrast were improved with *γ*–correction and contrast-limited adaptive histogram equalization. Reddy et al. [44] used texture histogram equalization. Foracchia et al. [45] and Leahy et al. [46] proposed methods based on the estimation of degradation features, such as luminance, contrast, or illumination to achieve enhancement. Kubecka et al. [47] proposed the optimization of parameters of the B-spline shading model using Shannon’s entropy. Mustafa et al. [48] proposed a normalization of the background image using a low pass filter and a gaussian filter. These methods are based on local pixel statistics, and applicable without prior learning from ground truth (GT) images. However, this also leads to limited adaptability or generalizability, depending on the complex degradation factors in the fundus image.

Many studies have also been proposed on fundus image enhancement using deep learning. Savelli et al. [49] devised a structurally serialized CNN for correcting illumination. Even with a simple CNN structure, information on degradation characteristics on the fundus image is adeptly inferred by understanding the relative context of the patch. Zhao et al. [50] proposed a GAN-based framework to enhance blurry fundus images. This GAN architecture does not require actual low–high-quality training image pairs, and is suitable when data is limited. However, the number of degradations that can be improved at one time is limited because the latent space in GAN is uninterpretable and unmanipulable.

Since deep learning-based methods require substantial training data, synthesized images can effectively supplement insufficient real training images [51]. Methods that model the degradation factors are thus relevant in this context. Hide [52] introduced an atmospheric scattering model to explain the formation of haze, and this was further developed by other studies [53, 54]. Xiong et al. [55] modeled a blurry fundus image, using the atmospheric scattering model, suggesting a method for estimating the transmission map and background illuminance. Shi et al. [56] applied *γ*–correction to the model and improved image illumination.

### CNN architectures with attention

Here, we review relevant CNN architectures to our customized attention-based CNN network. Attention within a CNN is an operation where the network learns to attend to particular feature values through adaptive scaling. Many attempts have been made to incorporate various attention mechanisms into networks [57, 58]. The network learns to scale local features through spatial attention. The network also learns to scale particular feature channels, corresponding to important image characteristics, through channel attention.

Oktay et al. [59] and Li et al. [60] proposed network structures that combined a spatial-attention module with U-Net [38]. These studies learned the relative importance of spatial between pixels of a feature map for performing segmentation of a target object in a medical image. Rundo et al. [61] confirmed the importance of channel-wise recalibration of the feature map in the segmentation task of MRI image, by inserting a Squeeze-and-Excitation (SE) module [62], which was a channel-attention within a U-Net. Studies also combined the spatial-attention and channel attention parallelly [63, 64] or sequentially [65]. Sun et al. [66] included a parallel spatial and channel attention structure in the skip connections between the encoder and decoder blocks in the U-Net. Zhao et al. [67] and Gu et al. [68] used a sequential spatial and channel attention structure. Zhao et al. [67] noted that a spatial-attention module was used at the network interface; whereas a channel-attention module was used to generate latent representations and reduce computational complexity. Gu et al. [68] placed channel-attention modules at every decoder block to learn to generate segmentation maps from the encoded latent representation.

## Methods

### Data preprocessing

#### Registration

**Fig 1:**
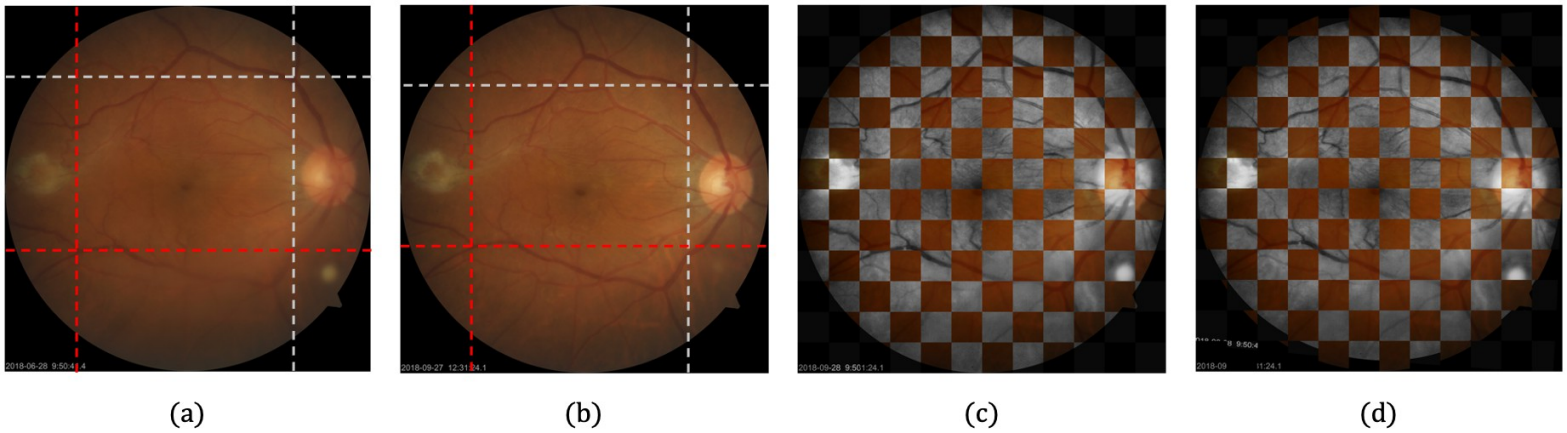
Registration for fundus photograph. (a) Low-quality (LQ) image before registration. (b) High-quality (HQ) image before registration. (c) Checkerboard image before registration with grayscale LQ image and color HQ image. (d) Checkerboard image after registration with grayscale LQ image and color HQ image. The vertical and horizontal dotted lines on (a) and (b) are crossing over singular points (where the blood vessel line diverges) that exist in common in LQ and HQ images.

Given that fundus image pairs for the same patient at different times are nonidentical due to the differences in camera viewpoint or patient pose, image registration is required to ensure the local correspondence of LQ and HQ images during network training. We used the SURF–PIIFD–RPM method, proposed by Wang et al. [69], using affine transformation or second-order polynomial transformation depending on the image, to perform robust alignment for the image with rotation and scale-invariant SURF feature points [70]. We manually annotated the corresponding points to guide the registration in the rare cases, where SURF key point matches were obtained incorrectly. Fig 1 shows the registration results of a sample image pair from the training dataset.

#### Patch generation

To adhere to the constraints in GPU memory, we used smaller patches of size 320 × 320 × 3, cropped from the original images. For training, we chose 5 patches around the macular, 10 patches around the crossing point of the vessels, and 5 patches randomly across the entire fundus image. We tested our network on non-overlapping tiled patches of the whole image.

#### Augmentation

We supplemented the limited number of images in our dataset using data augmentation. We considered five different augmentation factors: i) rotation, ii) linear interpolation, iii) blur, iv) haze, and v) illumination.

For rotation, we added three rotated versions of images with angles of 90°, 180°, and 270°. With the additional rotations, the network can learn rotation-agnostic features, such as vessels or macular patterns, which must be consistently enhanced, invariant to image orientation.

For linear interpolation, we generated new LQ images, *L*_*I*,*new*_ using linear interpolation between the LQ, *L*_*I*_ and HQ, *H*_*I*_ images as follows:

$$\begin{array}{c} L_{I,new}=\left(H_{I}-L_{I}\right)\lambda +L_{I}, \end{array}$$ (1)

where we assigned four different values for the scalar variable λ = (0.2, 0.4, 0.6, 0.8), which controls the degree of interpolation. This augmentation enables the network to consistently enhance images with intermediate qualities between the LQ and HQ images [71].

For the blur, we generated new LQ images *L*_*I*,*new*_ using Gaussian blur [72] as follows:

$$\begin{array}{c} L_{I,new}\left(x,y\right)=\Sigma_{i}\Sigma_{j}H_{I}\left(x-i,y-i\right)K\left(i,j\right), \end{array}$$ (2)

where *H*_*I*_ is the patch from the original HQ image, and *K* is a gaussian kernel for convolution. Here, we used a Gaussian blur kernel of size 5 × 5.

**Fig 2:**
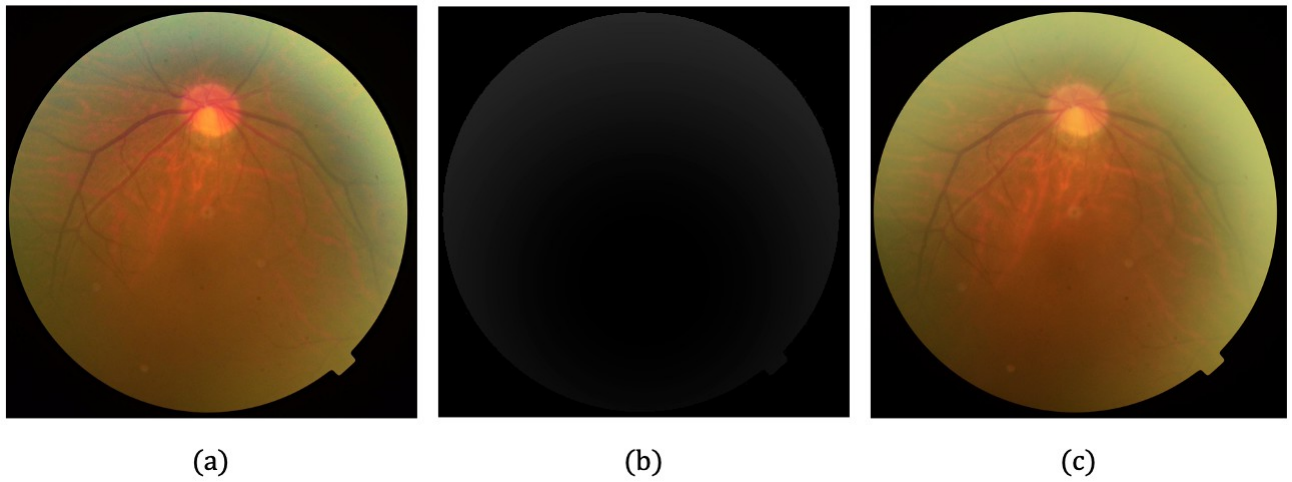
Synthesizing hazy image. (a) Original HQ image. (b) Manually crafted depth map. (c) Created hazy image.

For haze, we applied the atmospheric scattering model [52] to synthesize new LQ hazy images *L*_*I*,*new*_ assuming a homogeneous transmission map and several manually crafted depth maps *d*(*x*), as shown in Fig 2. This model is formulated as follows:

$$\begin{array}{c} L_{I,new}\left(x\right)=H_{I}\left(x\right)t\left(x\right)+\left(1-t\left(x\right)\right)A, \end{array}$$ (3)

where *t*(*x*) is the transmission map; *H*_*I*_(*x*) is the original HQ image, and *A* is the atmospheric light vector in the RGB domain. We can assume that the transmission map is homogeneous, and *t*(*x*) is represented as follows:

$$\begin{array}{c} t\left(x\right)=e^{-\beta d(x)}, \end{array}$$ (4)

where *β* is the medium extinction coefficient, and *d*(*x*) is the depth between the objects and the camera.

Finally, for illumination, we used *γ*–correction, which is a nonlinear transformation that adjusts the brightness of the image [56] to generate the unevenly illuminated LQ image *L*_*I*,*new*_. This model is formulated as follows:

$$\begin{array}{c} L_{I,new}\left(x,y\right)=H_{I}{\left(x,y\right)}^{\frac{1}{\gamma }}, \end{array}$$ (5)

where the *γ* value, in the range of 0 < *γ* < 1, darkens the image and simulates low illumination.

### Proposed network architecture

**Fig 3:**
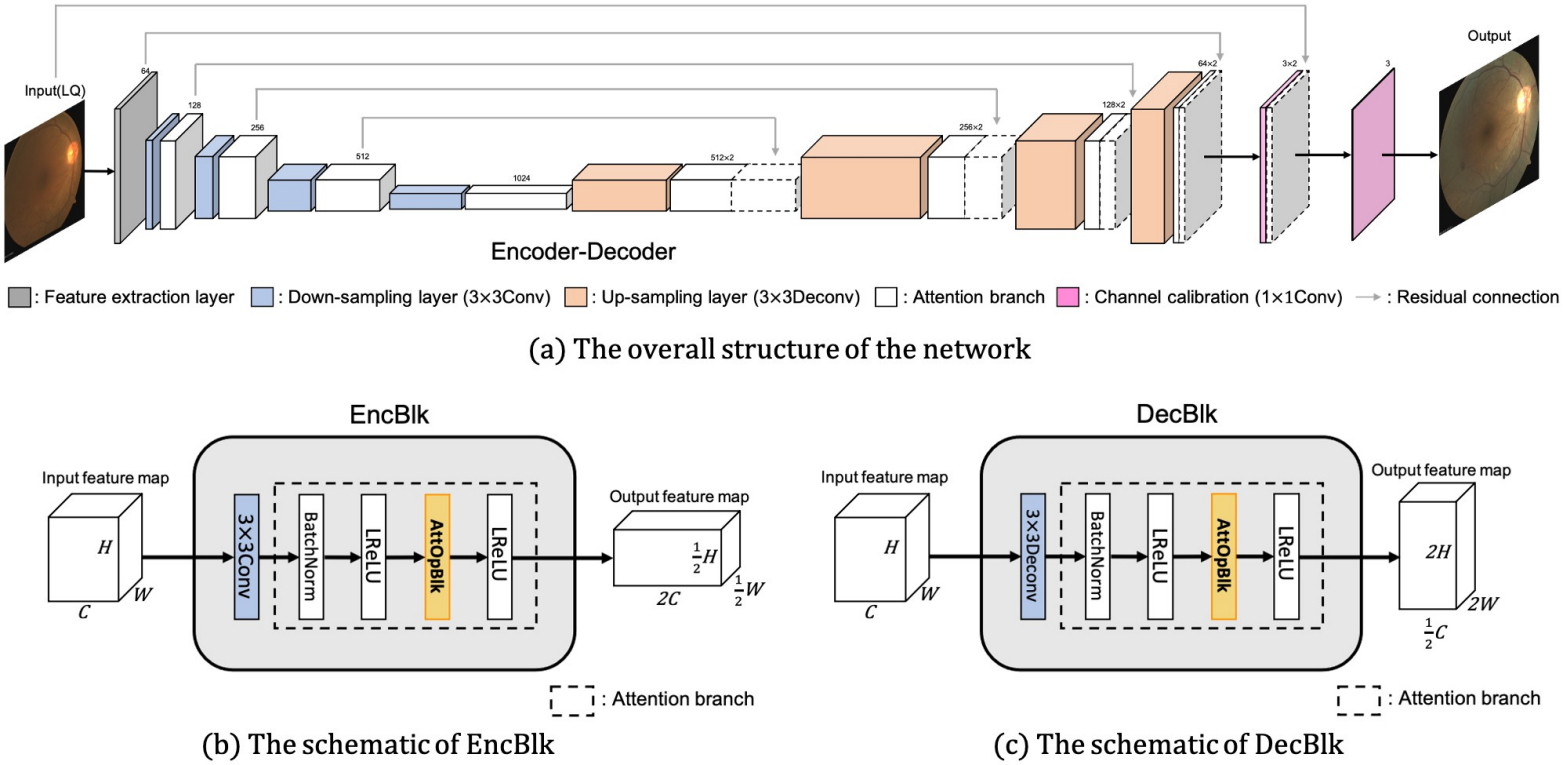
The overall architecture of the proposed method. The first half of the network encodes the fundus image to latent representation; whereas the second half decodes it again to reconstruct the enhanced fundus image. The whole symmetric network is trained in an end-to-end manner.

Our customized network is a convolutional neural network (CNN) with an encoder-decoder structure similar to U-Net [38], as depicted in Fig 3. While this structure has been found to work well for general image enhancement [73], we include an additional layer that incorporates parallel operations within a channel attention framework, so that specific aspects of the enhancement corresponding to the given image can adaptively emphasized.

The encoding and decoding blocks, denoted as EncBlks and DecBlks, respectively, have nearly identical structures, except for the first 3 × 3conv and 3 × 3deconv layers, because EncBlks must downscale the input size and DecBlks must upscale the downsampled input. We used the parallel layer and adaptive attention mechanisms to selectively apply suitable operations for the given input [74], as AttOpBlk.

**Fig 4:**
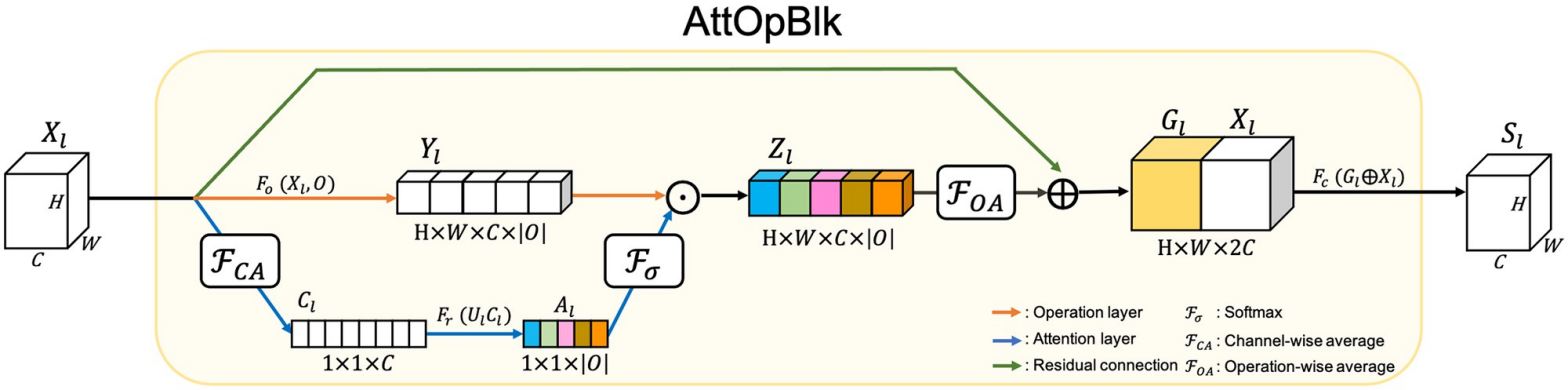
Structure of AttOpBlk. The attention vector *A*_*l*_ learned using the channel-wise average of the input feature map is multiplied with *Y*_*l*_, the result of applying the operations in the operation set on the input feature map. The attention layer can learn the optimal operation according to the degradation characteristics of the input feature map.

We applied five parallel operations in AttOpBlk: {1 × 1conv, 3 × 3conv, 5 × 5conv, 7 × 7conv, 3 × 3maxpool}, and a channel-wise attention layer to compute the attention weight, indicating the importance of each operation. The attention layer computes the attention weight through a 3-Layer-MLP with a channel-wise average of the input feature map and finds the optimal operation to be used in the corresponding EncBlk and DecBlk, considering various factors such as feature map size, degradation factors, the severity of degradation, and layer depth. As shown in Fig 4, at AttOpBlk *l*, the attention weight *A*_*l*_ is expressed as follows:

$$\begin{array}{c} A_{l}=F_{r}\left(U_{l}C_{l}\right), \end{array}$$ (6)

where $U_{l}\in R^{\left|O||C_{l}\right|}$ is the learnable matrix; |*O*| is the number of operations in the attention layer; *F*_*r*_ is the ReLU function, and *C*_*l*_ is the per-channel spatial average of input *X*_*l*_ as follows.

$$\begin{array}{c} C_{l,c}=\frac{1}{H\times W}\sum_{i=1}^{\left|H\right|}\sum_{j=1}^{\left|W\right|}X_{l}\left(i,j,c\right), \end{array}$$ (7)

where *H* and *W* refer to the height and width of the input feature map *X*_*l*_, and *c* denotes the channel of the input feature map *X*_*l*_. We used the per-channel average as the input of the channel-wise attention layer because the absolute intensity of the pixel map of the input feature has a significant impact in determining the degradation factor and its severity.

Subsequently, vector *A* is normalized into $\bar{A}$ such that the sum of the elements of attention weight is 1, and *Z*_*l*_ is the result of the element-wise multiplication of $\bar{A}$ and *Y*_*l*_, the results of applying each operation in the operation set to the input feature map of the layer. This process is formulated as follows:

$$\begin{array}{c} {\bar{A}}_{l,i}=\frac{e^{A_{l,i}}}{\sum_{j}e^{A_{l,j}}}, \end{array}$$ (8)

$$\begin{array}{c} Z_{l}=Y_{l}⊙{\bar{A}}_{l}, \end{array}$$ (9)

where ⊙ denotes the element-wise multiplication, and *Y*_*l*_ = *O*(*X*_*l*_) is the result of applying operations in the operation set on the input feature map *X*_*l*_.

The input feature map *X*_*l*_ is concatenated with the sum of the *Z*_*l*_ to retain the knowledge learned in the previous layer, This connection is also interpreted as a residual connection [75] between the input and output of the layer that enables the gradient to be propagated into the input of the layer through backpropagation. Finally, a 1 × 1conv operation is placed at the end of the layer to adjust the channel of the output feature map of the AttOpBlk, and the output of the AttOpBlk *l*, *S*_*l*_ is computed as follows:

$$\begin{array}{c} S_{l}=F_{c}(G_{l}⊕X_{l}), \end{array}$$ (10)

$$\begin{array}{c} G_{l}=\sum_{o=1}^{\left|O\right|}Z_{l,o}, \end{array}$$ (11)

where |*O*| is the number of operations in the operation set; *F*_*c*_ denotes 1 × 1 convolution, and ⊕ denotes channel-wise concatenation of two matrices.

As shown in Fig 3, the entire network is structured following a composition of EncBlks and DecBlks. The width and height of the feature map are downsampled from the image by 2^4^, and the feature dimension becomes 2^10^ after the encoding portion in the first half of the network. For example, a latent feature representation of size 20 × 20 × 1024 results from an input image of size 320 × 320 × 3. In the decoding portion of the second half of the network, the latent features are upsampled and reconfigured to become an output of size 320 × 320 × 3, identical to the input.

To train the network, we use the following loss function:

$$\begin{array}{c} L=\frac{1}{N}\sum_{i=1}^{N_{batch}}\|\hat{y_{i}}-y_{i}{\|}_{1}+\frac{\lambda }{2}\|W_{net}{\|}^{2}, \end{array}$$ (12)

where *y* is the output of the network; $\hat{y}$ is the reference image; *N*_*batch*_ is the number of images in the minibatch, and *W*_*net*_ is the weight parameters of the network. The first term is the pixelwise difference term to supervise the network output to be similar to the ground truth (the HQ image), while the second term is the L2 norm for the trainable weights of the network, which is a commonly used regularization term [76]. We used the L1 distance for the pixel-wise difference. Unlike other tasks, L2 distance may over-penalize the values in pixels with uneven illumination [77], given that our training dataset contained numerous dark LQ images and bright HQ image pairs. The parameter λ, set at λ = 0.1, controls the relative importance between the two terms.

### Datasets

We sampled the training dataset comprising 1068 pairs of LQ and HQ fundus photographs of patients, acquired from the Kangbuk Samsung Hospital Ophthalmology Department (KBSMC) between 2017 and 2019, and denoted this as the KBSMC dataset. LQ images were taken either in the health screening process or from a preoperative examination. Corresponding HQ images are from the same patient, acquired after pupil dilation or surgery, from which accurate diagnosis can be achieved.

**Fig 5:**
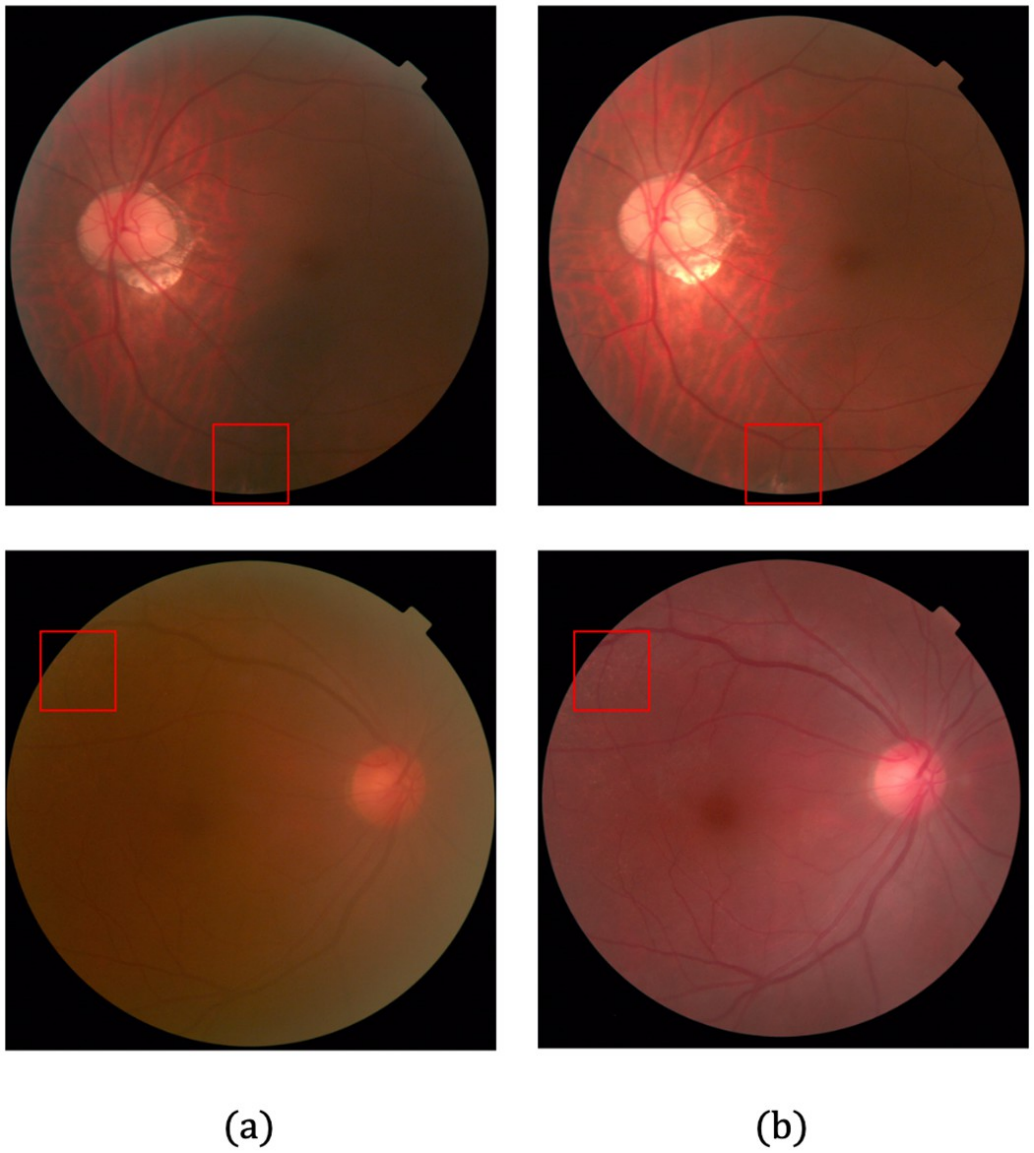
Examples of enabling diagnosis. Each row depicts images sampled from LQ and HQ samples, where lesions that were unnoticeable in LQ image are clarified in the corresponding HQ image. (a) LQ images. (b) HQ images corresponding to (a).

In Fig 5, we depict two examples from the KBSMC dataset where improvements in image quality facilitate better diagnosis. We can observe regions (in the red boxes) where lesions become visible in the HQ images (small round hole and drusen for the first and second example, respectively). The majority of eye diseases are found in the peripheral region of the retina. Thus, these examples show how the peripheral region of the retinal fundus image is as important as the central field, and how well our KBSMC dataset is designed to train our model for various degradations on the retinal fundus image.

Fundus photographs were taken with various manufacturers’ nonmydriatic fundus cameras, including TRC-NW300, TRC-50IX, TRC-NW200 and TRC-NW8 (Topcon, Tokyo, Japan), CR6-45NM and CR-415NM (Canon, Tokyo, Japan), and VISUCAM 224 (Carl Zeiss Meditec, Jena, Germany). Digital images of the fundus photographs were analyzed using a picture archiving and communication system (INFINITT, Seoul, Korea). All images were of a resolution of 3600 × 3600.

For evaluation, we constructed a test dataset from images, acquired from the ophthalmology department of Seoul National University Hospital (SNUH), denoted as the SNUH dataset. This dataset comprised 68 pairs of fundus photographs collected before and after cataract surgery, of which 29 (42.6%) of the pre-surgery LQ images were ungradable. Here, all images were of a resolution of 2400 × 2400.

Since we were unable to share the private datasets due to privacy issues, we also used the publicly available DRIVE [78], STARE [79], CHASE_DB1 [80] and DIARETDB1 [81] datasets, comprising 40, 397, 28, and 89 images, respectively, as additional test datsaets. We chose the DRIVE [78], STARE [79] and CHASE_DB1 [80] datasets because they are commonly used by studies, focusing on retinal fundus images and the evaluation of retinal vessel segmentation methods. The DIARETDB1 [81] dataset was chosen because many of its images have poor illumination and thus are suitable for the proposed method.

This study adhered to the tenets of the Declaration of Helsinki, and the protocol was reviewed and approved by the Institutional Review Boards (IRB) of Kangbuk Samsung Hospital (No. KBSMC 2019-08-031) and Seoul National University Hospital (C-2007-003-1137). Our study is a retrospective of medical records, and our data were fully anonymized before processing. The IRB waived the requirement for informed consent.

## Experimental results

### Evaluation settings and metrics

Training was performed using the entire KBSMC dataset, whereas testing was performed on the external SNUH dataset and publicly available DRIVE [78], STARE [79], CHASE_DB1 [80], and DIARETDB1 [81] datasets. Additionally, we performed five-fold cross-validation on the KBSMC dataset to serve as a reference when there is no domain shift.

We used three metrics to assess the quality of the enhanced image and to evaluate the proposed framework: i) PSNR [82], ii) SSIM [83], iii) *r* (linear index of fuzziness) [84, 85]. For the SNUH dataset, we also measure the proportion of ungradable fundus images before and after the enhancement process.

Both PSNR and SSIM are reference metrics, used to measure the quality when compared with the reference GT. PSNR may not correspond to human intuition of overall image quality given that PSNR is based solely on the pixel-wise mean-squared error (MSE) between the output image and GT. For example, a blurred output may lead to a lower MSE than a similar but slightly misaligned texture for high-frequency texture details [86]. Thus, we also used SSIM, which measures degradation as the relative change in perceived structural information. *r* is independent of the GT and can be measured solely from the output image. We primarily applied this metric to the public datasets that lacked the GT HQ images to serve as references. For PSNR or SSIM, higher values indicate that the enhanced image is closer to the GT image; whereas, for *r*, a lower value indicates a less noisy image and thus better performance. (This metric is originally denoted as *γ* by [84, 85]. However, we denote this as *r* to avoid confusion with the *γ* in *γ*-correction).

To measure ungradable images, we define LQ images as ungradable following Fleming et al. [87] as: i) Images in which the third-generation branches cannot be identified within one optic disc diameter of the macular. ii) Images with various artifacts. iii) Images in which at least one of the macular, optic disc, superior temporal arcade, or inferior temporal arcade are incomplete. iv) Images in which the diagnosis cannot be obtained because of the degradation.

We also conducted a comparative evaluation, where we presented the PSNR, SSIM, and *r* results of three different algorithms, developed by Zhou et al. [42], Gaudio et al. [88], and Dai et al. [89], respectively along with the *P*-values of the proposed method.

### Evaluation of private datasets

Table 1 shows the quantitative comparative evaluations of the KBSMC and SNUH test datasets, demonstrating that the proposed method achieves the best results for both datasets. When compared with the original input LQ image, the proposed method achieves an average increase of 8.74 **dB** in PSNR, a 0.29 increase in SSIM, and a 0.51 decrease in *r* values for the KBSMC test dataset, a 7.26 **dB** increase in PSNR, 0.20 increase in SSIM, and 0.29 decrease in *r* values for the SNUH dataset. Furthermore, when compared with the method with the next best result, the proposed method achieves an average of 5.15 **dB** increase in PSNR, a 0.03 increase in SSIM, and a 0.07 decrease in *r* values for the KBSMC test dataset, a 4.31 **dB** increase in PSNR, 0.04 increase in SSIM, and 0.17 decrease in *r* values for the SNUH dataset.

**Table 1:** Quantitative comparison on private datasets.

	KSH test set (*n* = 100)			SNUH test set (*n* = 68)		
Methods	PSNR(dB) ↑ (*P* value)	SSIM ↑ (*P* value)	*r* ↓ (*P* value)	PSNR(dB) ↑ (*P* value)	SSIM ↑ (*P* value)	*r* ↓ (*P* value)
Input LQ image	21.83 ± 2.18	0.61 ± 0.08	0.83 ± 0.07	20.02 ± 1.87	0.65 ± 0.1	0.57 ± 0.14
Zhou et al. [42]	23.81 ± 1.25 (< 0.001)	0.87 ± 0.04 (< 0.001)	0.68 ± 0.16 (< 0.001)	19.28 ± 1.73 (< 0.001)	0.79 ± 0.02 (< 0.001)	0.59 ± 0.12 (< 0.001)
Gaudio et al. [88]	19.23 ± 0.99 (< 0.001)	0.67 ± 0.12 (< 0.001)	0.39 ± 0.06 (< 0.001)	21.4 ± 0.97 (< 0.001)	0.7 ± 0.06 (< 0.001)	0.45 ± 0.12 (< 0.001)
Dai et al. [89]	25.42 ± 1.97 (< 0.001)	0.83 ± 0.02 (< 0.001)	0.43 ± 0.13 (< 0.001)	22.97 ± 1.48 (< 0.001)	0.81 ± 0.05 (< 0.001)	0.49 ± 0.13 (< 0.001)
**Ours**	**30.57** ± **1.67**	**0.90** ± **0.03**	**0.32** ± **0.11**	**27.28** ± **0.76**	**0.85** ± **0.03**	**0.28** ± **0.12**

Values are mean ± standard deviation.

For PSNR and SSIM, larger values, and for *r*, smaller values indicate better performance, respectively.Bold values denote the most effective method, corresponding to each evaluation metric.

The *P*-value represents the statistical significance of our enhancement approach, compared with other methods.

**Fig 6:**
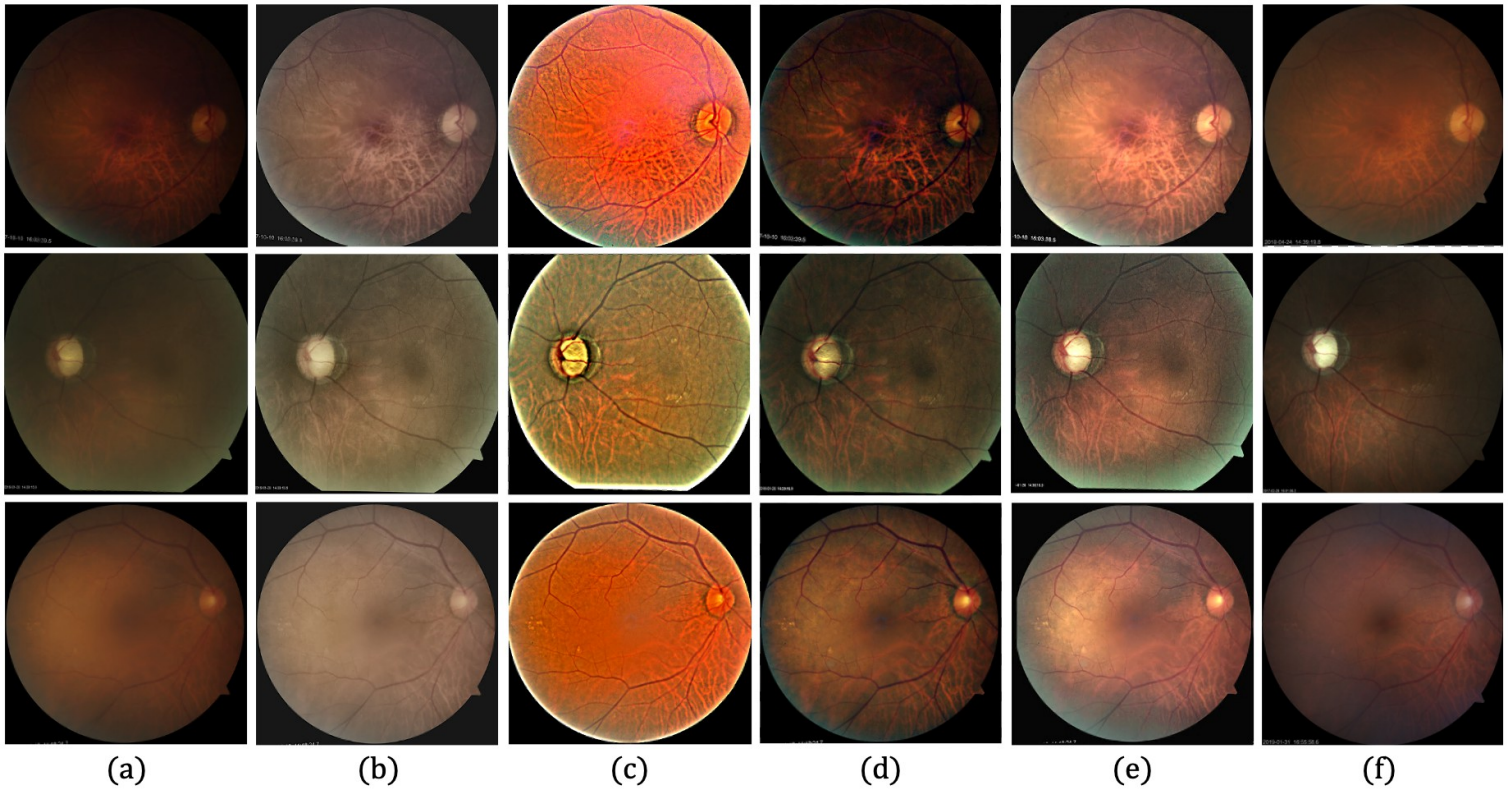
Qualitative comparison of three LQ image samples from the KBSMC private test dataset. (a) Input LQ images, and results using the methods of (b) Zhou et al. [42], (c) Gaudio et al. [88], (d) Dai et al. [89], (e) the proposed method, and (f) GT of (a).

**Fig 7:**
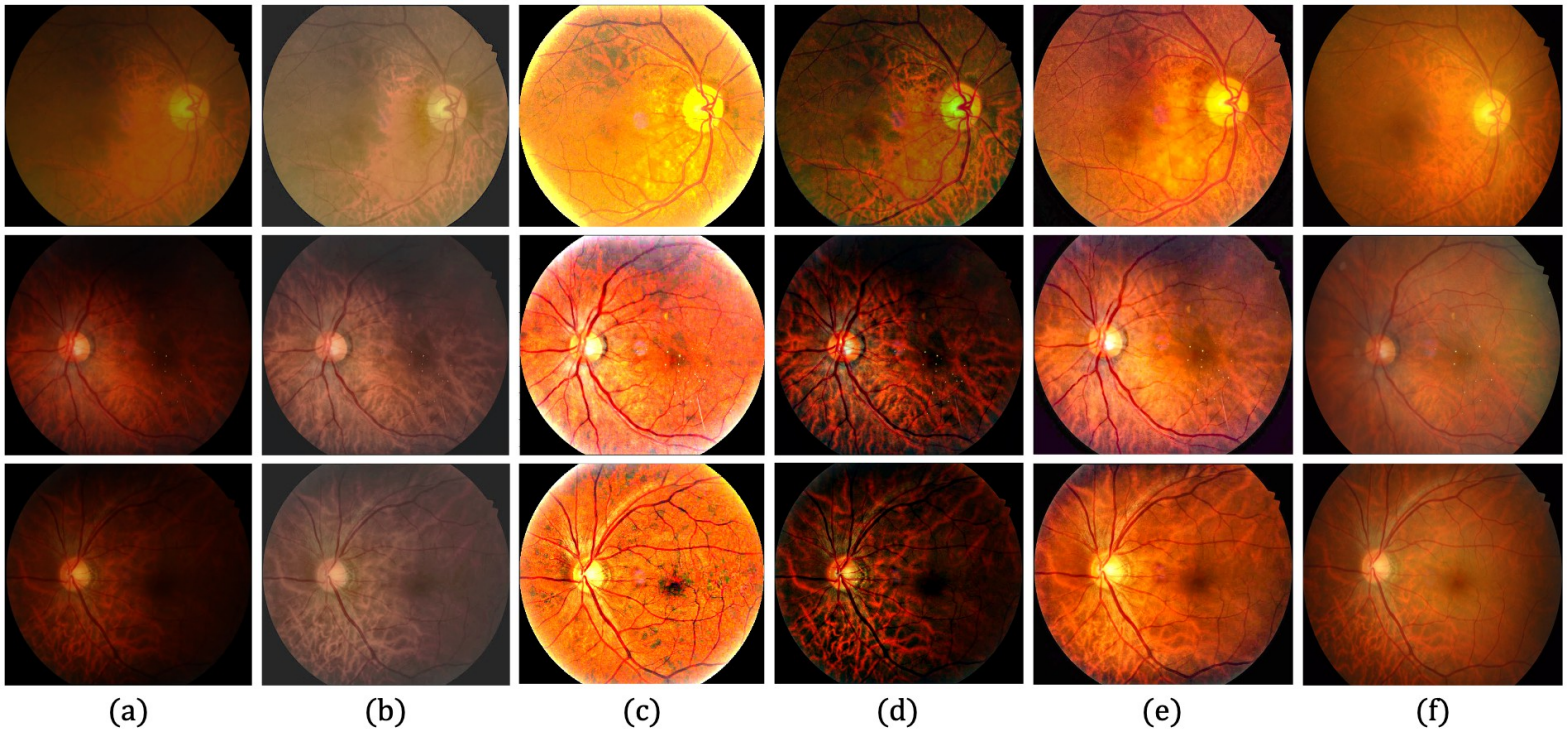
Qualitative comparison of three LQ image samples from SNUH private test dataset. (a) Input LQ images, and results using the methods of (b) Zhou et al. [42], (c) Gaudio et al. [88], (d) Dai et al. [89], (e) the proposed method, and (f) GT of (a).

Fig 6 provides qualitative comparisons of sample images with the KBSMC test dataset. Based on a visual comparison with the HQ GT, the proposed method seems to recover more of the characteristics lost from the degradation compared with those recovered by other methods. Fig 7 shows the qualitative comparisons of the sample images with the SNUH test set.

We also compared the change in the proportion of ungradable fundus photographs with the SNUH dataset, based on our method. Among the 68 images from the SNUH datsaet, the ungradable images were reduced from 29 (42.6%) to 18 (26.4%), with a statistical significance of *P* = 0.012, computed from McNemar’s test.

### Evaluation of public datasets

We applied our trained model to four public datasets (DRIVE [78], STARE [79], CHASE_DB1 [80] and DIARETDB1 [81]) to demonstrate how effectively the proposed data augmentation method synthesized various degradations, and how our pre-trained model improved the LQ image, sampled from the out-of-distribution datasets.

**Fig 8:**
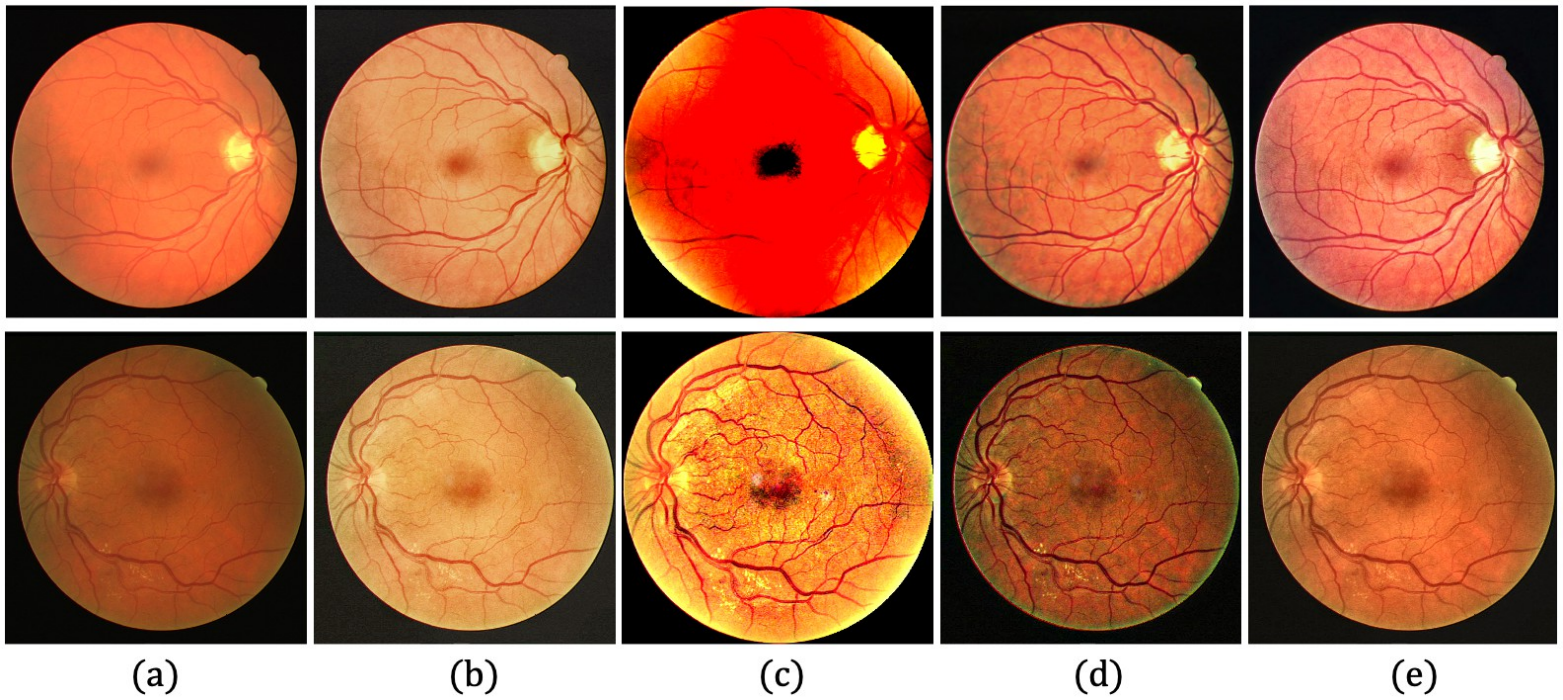
Qualitative comparison of two LQ image samples from the DRIVE database. (a) Input LQ images, and results using the methods of (b) Zhou et al. [42], (c) Gaudio et al. [88], (d) Dai et al. [89], and (e) the proposed method.

Table 2 shows the quantitative evaluation of each dataset, based on the average *r* values, revealing whether *P*-values are within the level of statistical significance of 0.001. Although the proposed method produces the lowest *r* values for the DRIVE [78], STARE [79] and CHASE_DB1 [80] datasets, it produces a higher *r* value than the Gaudio et al. [88] method for the DIARETDB1 [81] dataset. This could be associated with the characteristics of Gaudio et al. [88] method, which maximizes the underlying pattern of the fundus image after amplifying the pixel color. However, the image may be unrealistic after drastically altering the appearance of the original image. Figs 8–Fig 11 provide qualitative comparisons of sample images from the DRIVE [78], STARE [79], CHASE_DB1 [80] and DIARETDB1 [81] datasets, respectively. The proposed method improves the image, makes its content visible more clearly, and minimizes unwanted changes.

**Fig 9:**
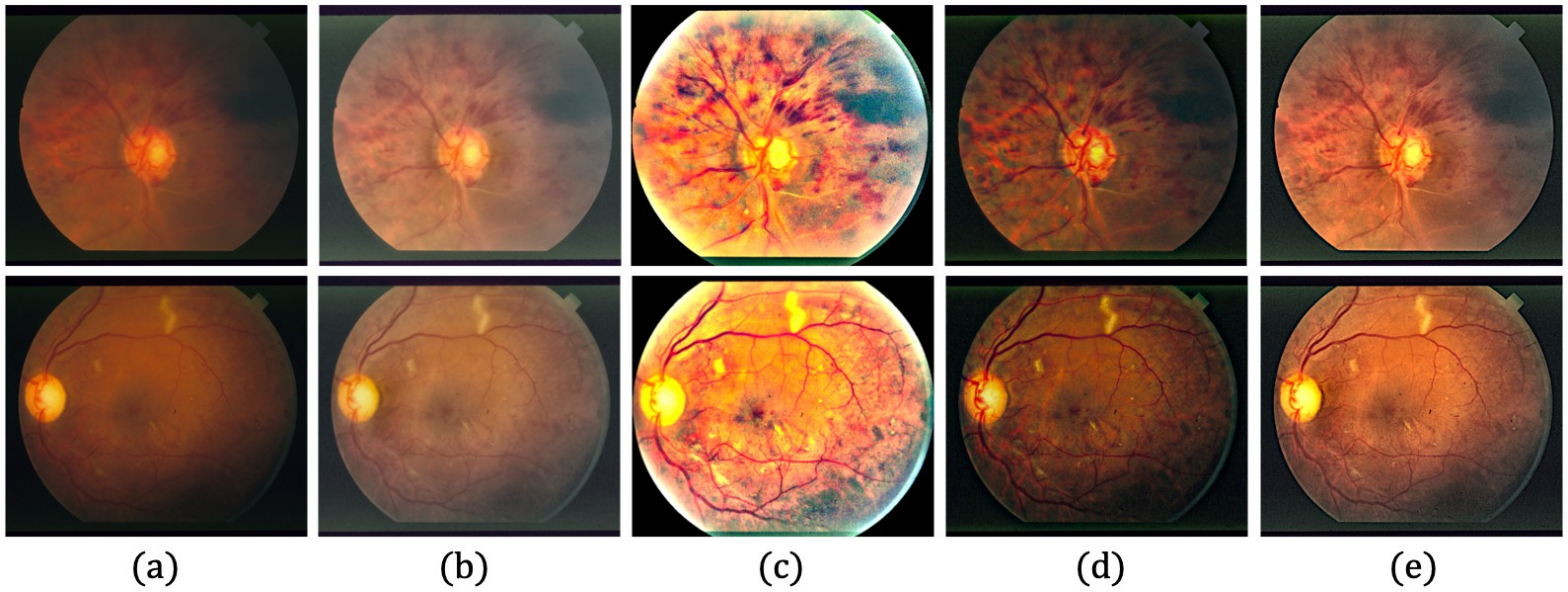
Qualitative comparison of two LQ image samples from the STARE database. (a) Input LQ images, and results using the methods of (b) Zhou et al. [42], (c) Gaudio et al. [88], (d) Dai et al. [89], and (e) the proposed method.

**Fig 10:**
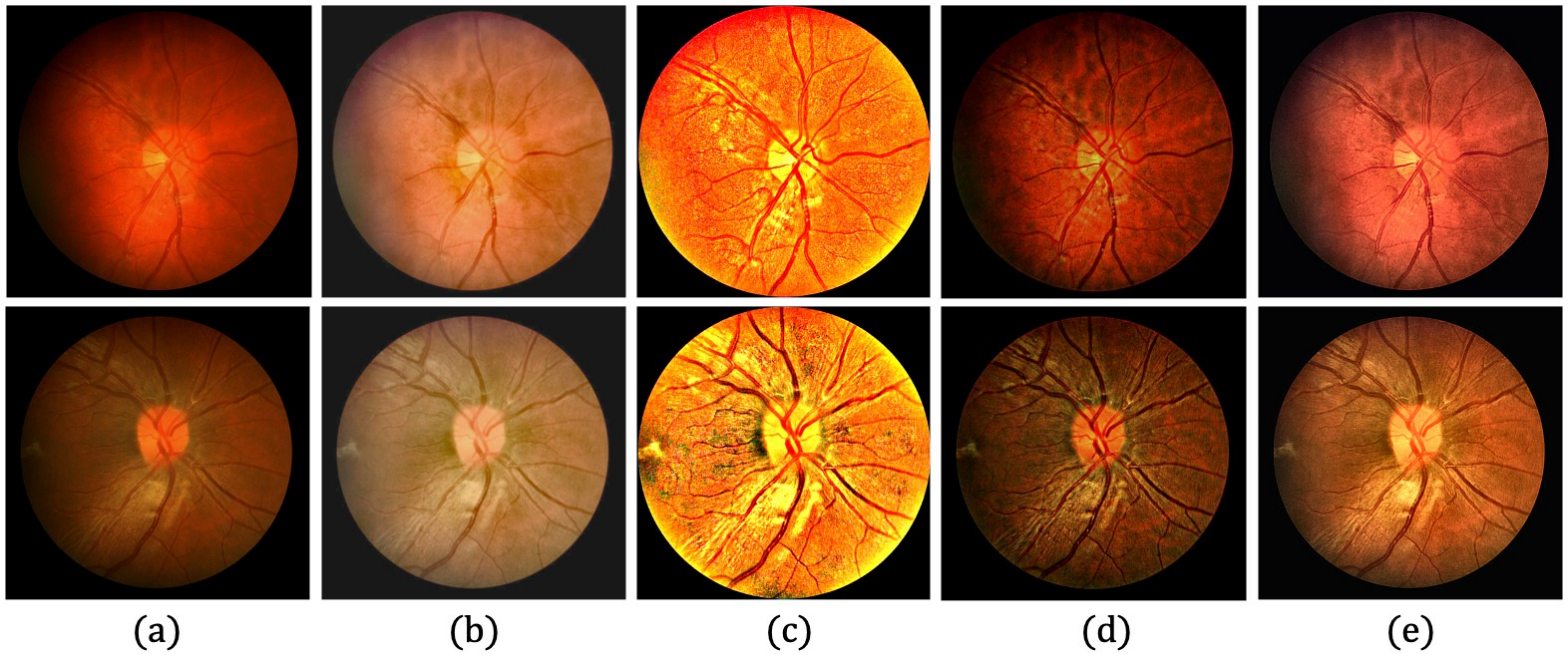
Qualitative comparison of two LQ image samples from the CHASE_DB1 database. (a) Input LQ images, and results using the methods of (b) Zhou et al. [42], (c) Gaudio et al. [88], (d) Dai et al. [89], and (e) the proposed method.

**Fig 11:**
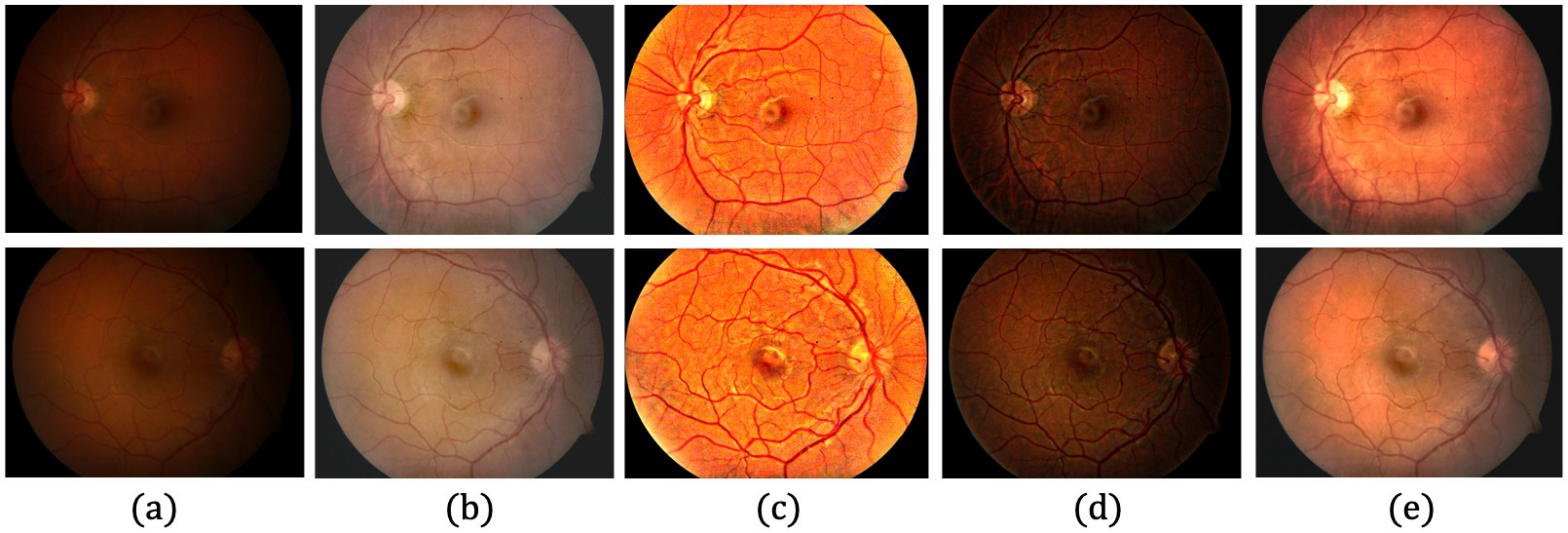
Qualitative comparison of two LQ image samples from the DIARETDB1 database. (a) Input LQ images, and results using the methods of (b) Zhou et al. [42], (c) Gaudio et al. [88], (d) Dai et al. [89], and (e) the proposed method.

**Table 2:** Quantitative comparison on public datasets.

	DRIVE database (*n* = 40)		STARE database (*n* = 397)		CHASE_DB1 database (*n* = 28)		DIARETDB1 database (*n* = 89)	
Methods	*r* ↓	*P* value	*r* ↓	*P* value	*r* ↓	*P* value	*r* ↓	*P* value
Input LQ image	0.43 ± 0.08		0.47 ± 0.11		0.27 ± 0.09		0.28 ± 0.12	
Zhou et al. [42]	0.59 ± 0.05	< 0.001	0.57 ± 0.06	< 0.001	0.51 ± 0.09	< 0.001	0.63 ± 0.07	< 0.001
Gaudio et al. [88]	0.36 ± 0.09	< 0.001	0.49 ± 0.06	< 0.001	0.51 ± 0.12	< 0.001	**0.17** ± **0.08**	< 0.001
Dai et al. [89]	0.35 ± 0.04	< 0.001	0.40 ± 0.09	< 0.001	0.22 ± 0.11	< 0.001	0.59 ± 0.02	< 0.001
**Ours**	**0.24** ± **0.01**		**0.35** ± **0.05**		**0.21** ± **0.06**		0.23 ± 0.01	

Values are mean ± standard deviation.

Smaller *r* values indicate better performance.

Bold values denote the most effective method, corresponding to each evaluation metric.

The *P*-value represents the statistical significance of our enhancement approach, compared with other methods.

### Implementation details

For the hyperparameters, we used a mini-batch size of 16, an initial learning rate of *α* = 0.01 and decay rate of 0.9, as shown by [75] for 1000 epochs, each of which has approximately 300 iterations.

For comparative evaluations of three algorithms, we implemented our version based on the Zhou et al. [42] and Dai et al. [89] methods, following their descriptions of network architecture and hyperparameter settings. Moreover, we used the official implementation of Gaudio et al. [88] with the sA + sB + SC+ sX option.

Each experiment with different datasets using our CNN-based network is performed on a single NVIDIA GeForce GTX 2080Ti GPU, which takes about 0.91 second per 320 × 320 × 3 scaled image, while the three algorithms developed by Zhou et al. [42], Gaudio et al. [88], and Dai et al. [89] were evaluated on a single Intel Xeon Gold 6248R CPU.

Statistical analysis was conducted using SPSS 24 (IBM SPSS Statistics 24, IBM Corporation, Armonk, NY, USA).

## Discussions

### Limitations

The proposed image enhancement framework is beneficial for most ungradable fundus images. However, two main limitations are identified that must be addressed. First is the accuracy of GT images. Although all corresponding LQ and HQ fundus photograph pairs are from the same patients, factors that can be detrimental when determining the GT fundus image are the time interval between image acquisitions, the differences in positions or angles, inconsistent alignment between LQ and HQ fundus images after registration, and ungradable images or images with unknown diagnoses. We addressed these issues by i) minimizing the time interval between image pair acquisitions, ii) attaining accurate registration using the SURF–PIIFD–RPM method [69], and iii) using fundus images with a confirmed ophthalmic diagnosis, following a dilated fundus examination conducted by ophthalmologists.

**Fig 12:**
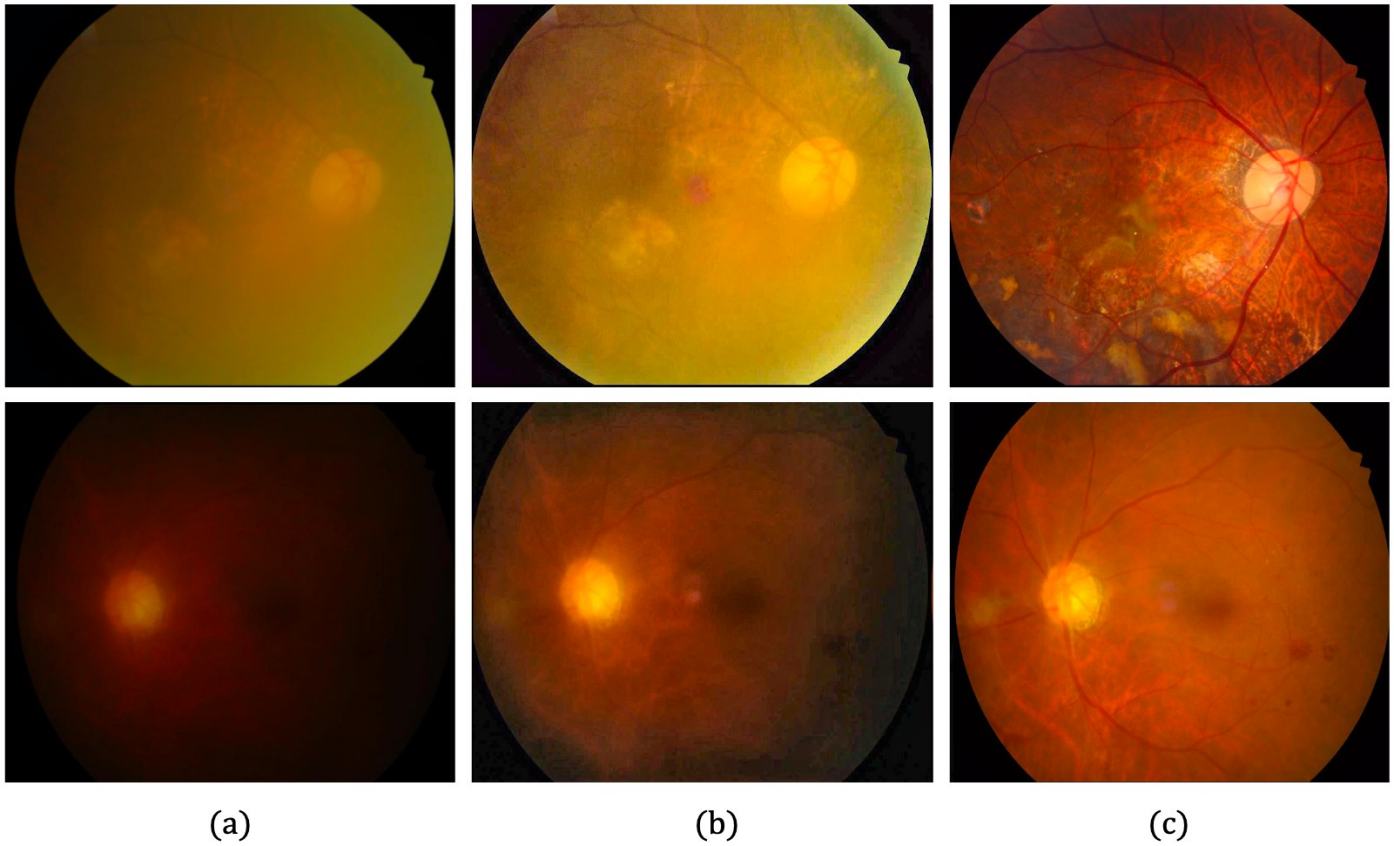
Failure cases. (a) Input LQ image. (b) Enhancement result of (a). (c) Original HQ image.

The disparity in the training and test datasets’ characteristics or the domain shift between datasets is the second limitation. The PSNR and SSIM values for the SNUH test dataset, in which the training and test datasets are from different domains, are lower than those of the KBSMC test dataset, which is from the same domain with training samples. In Fig 12, we show the failure cases of the SNUH test dataset. These examples illustrate the limitations of our image enhancement framework because the input image has a very low illumination. We note that the SNUH test set contains more severe cases of ungradable fundus images compared with the KBSMC dataset. Thus, the proposed framework may not work for test images with degradations, with different or more severe than those of the training images.

### Clinical application

Experimental results on the SNUH dataset demonstrate that the proposed method can be used to reduce ungradable images. Thus, we plan to apply our method to images acquired during health screening. Our goal is to reduce unnecessary re-examinations and save the patient’s time, money, and effort. Our framework can increase the diagnostic accuracy for LQ fundus photography, crucial for the ophthalmologist.

**Fig 13:**
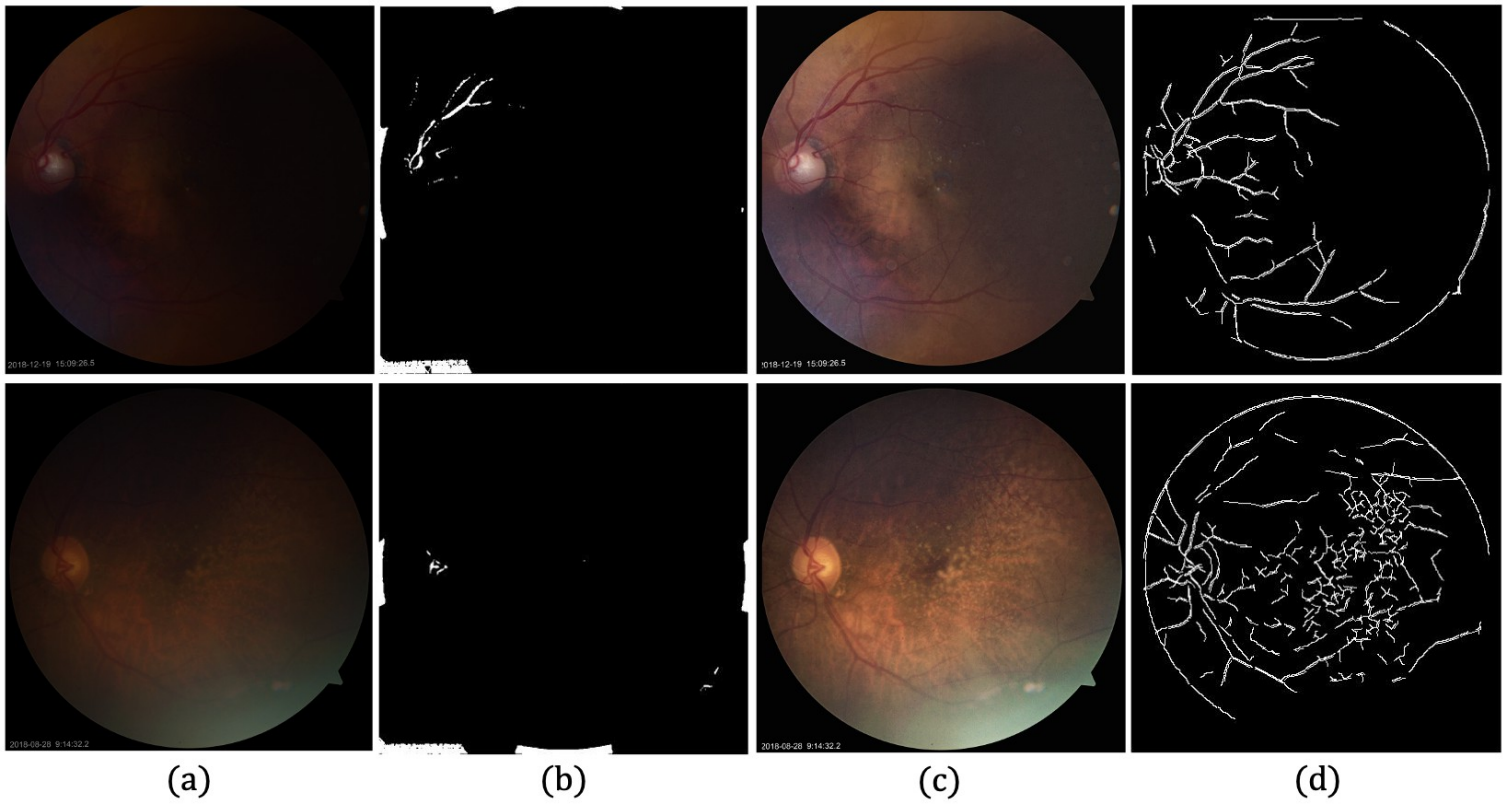
Segmentation of both LQ image and enhanced image. (a) Input LQ image. (b) Segmentation result corresponding to (a). (c) Enhancement result of (a). (d) Segmentation results corresponding to (c).

Our framework can also be used as a preprocessing step in other automated tasks, such as retinal vessel segmentation. Thus, the clarity of retinal blood vessels improves considerably after enhancement, as well as the results of vessel segmentation. Fig 13 depicts examples where the vessel segmentation are improved, using the iterative pixel thresholding method [90]. Two sampled ungradable fundus images and corresponding segmentation results also improved after enhancement.

## Conclusion

This study proposed a comprehensive framework for deep learning image enhancement of fundus images, comprising dataset collection, data augmentation, and customized network architecture. Pairs of LQ with many image degradation factors and corresponding HQ images were collected under a protocol, including clinical diagnosis by ophthalmologists and detailed analysis of the enhancement effect on pathological features within the fundus images. Based on our novel dataset, we proposed an optimal CNN structure for retinal fundus image enhancement that could effectively handle complex degradation factors with an attention module. The proposed framework was evaluated on internal and external validation datasets, as well as on DRIVE [78], STARE [79], CHASE_DB1 [80] and DIARETDB1 [81] databases. Among various poor image etiologies, our study provides a significant improvement in reducing the proportion of ungradable fundus photographs. Overall, our work is expected to have a clinical impact by lowering the rate of re-examinations among patients and by improving the accuracy of diagnosis.
